# BLOC-2 subunit HPS6 deficiency affects the tubulation and secretion of von Willebrand factor from mouse endothelial cells

**DOI:** 10.1016/j.jgg.2016.09.007

**Published:** 2016-12-20

**Authors:** Jing Ma, Zhe Zhang, Lin Yang, Janos Kriston-Vizi, Daniel F. Cutler, Wei Li

**Affiliations:** a Center for Medical Genetics, Beijing Children's Hospital, Capital Medical University, Beijing Pediatric Research Institute, MOE Key Laboratory of Major Pediatric Disease Research, Beijing 100045, China; b MRC Laboratory for Molecular Cell Biology, University College of London, London WC1E 6BT, UK; c State Key Laboratory of Molecular Developmental Biology, Institute of Genetics and Developmental Biology, Chinese Academy of Sciences, Beijing 100101, China; d Center of Alzheimer's Disease, Beijing Institute for Brain Disorders, Beijing 100069, China

**Keywords:** Hermansky-Pudlak syndrome, Weibel-Palade bodies, von Willebrand factor, Biogenesis of lysosome-related organells complex, Hemostasis

## Abstract

Hermansky-Pudlak syndrome (HPS) is a recessive disorder with bleeding diathesis, which has been linked to platelet granule defects. Both platelet granules and endothelial Weibel-Palade bodies (WPBs) are members of lysosome-related organelles (LROs) whose formation is regulated by HPS protein associated complexes such as BLOC (biogenesis of lysosome-related organelles complex) -1, -2, -3, AP-3 (adaptor protein complex-3) and HOPS (homotypic fusion and protein sorting complex). Von Willebrand factor (VWF) is critical to hemostasis, which is stored in a highly-multimerized form as tubules in the WPBs. In this study, we found the defective, but varying, release of VWF into plasma after desmopressin (DDAVP) stimulation in HPS1 (BLOC-3 subunit), HPS6 (BLOC-2 subunit), and HPS9 (BLOC-1 subunit) deficient mice. In particular, VWF tubulation, a critical step in VWF maturation, was impaired in HPS6 deficient WPBs. This likely reflects a defective endothelium, contributing to the bleeding tendency in HPS mice or patients. The differentially defective regulated release of VWF in these HPS mouse models suggests the need for precise HPS genotyping before DDAVP administration to HPS patients.

## 1 Introduction

Weibel-Palade bodies (WPBs) are secretory organelles in endothelial cells. They are large elongated rod shaped granules surrounded by a limiting membrane, with an internal tubular structure visible by electron microscopy (EM) (Weibel and Palade, 1964). Bioactive molecules that function in hemostasis, inflammation, angiogenesis and wound healing are stored in WPBs, and can be released by exocytosis in response to endothelial activation (Rondaij et al., 2006, Metcalf et al., 2008). The hemostatic factor von Willebrand factor (VWF) is the major content protein of WPBs. VWF has a highly complex biosynthesis, beginning with dimerization in the ER (endoplasmic reticulum), and subsequent formation of VWF tubules, allowing for multimerization and propeptide cleavage in a process that begins in the trans-Golgi network (TGN) and continues during subsequent maturation (Fowler et al., 1985, Vischer and Wagner, 1994, Purvis and Sadler, 2004, Huang et al., 2008, Springer, 2011). At secretagogue-triggered exocytosis, the VWF multimer unfurls into the flowing plasma as a long filament, highly active in platelet recruitment. VWF tubulation and multimerization as well as the length of WPB are essential to the formation of long platelet-catching strings (Michaux et al., 2006, Nightingale et al., 2009, Ferraro et al., 2014).

WPBs undergo complex multi-stage processes during their initial biogenesis and maturation stage (Metcalf et al., 2008, Nightingale and Cutler, 2013). WPB initial formation at the TGN is dependent on an AP-1 (adaptor protein complex-1)/clathrin coat that is essential to VWF folding into tubules and thus generation of elongated WPBs (Lui-Roberts et al., 2005). While some WPB components, such as P-selectin and IL6, are recruited during initial formation of immature electron-lucent WPBs with relatively few disorganized VWF tubules at the TGN, others are delivered to mature electron-dense WPBs with highly-ordered VWF tubules. For example, the P-selectin co-factor CD63 is delivered to mature WPBs in an AP-3-dependent mechanism, and Rab27a is also found on WPBs only late during their biogenesis when they tend to accumulate near the cell periphery (Hannah et al., 2003, Romani de Wit et al., 2003, Harrison-Lavoie et al., 2006, Zenner et al., 2007, Valentijn et al., 2008, Valentijn et al., 2011, Berriman et al., 2009). Further, VWF defects in forming tubules or in multimerization can cause misshaped WPBs (Michaux et al., 2003, Michaux et al., 2006), and interference with cellular machinery including aftiphilin or Rab27a can also cause defective multimerization of VWF (Nightingale and Cutler, 2013). The close relationships between WPB shape and VWF folding and multimerization are clear. However, the targeting of other WPB components, the cytoplasmic machinery that supports WPB formation, and the regulatory mechanisms controlling WPB biogenesis and exocytosis still require much further investigation.

The importance of such studies can be inferred from the fact that VWF unable to form high molecular weight (MW) multimers is one cause of von Willebrand disease (VWD), the most common inherited bleeding disorder (Sadler et al., 2000), yet elevated levels of highly-multimerized (HM) VWF in the blood can lead to vascular occlusions, as in thrombotic thrombocytopenic purpura (TTP) (Levy et al., 2001). Results from animal studies suggest that VWF deficiency is athero-protective, and in general, VWF multimerization is closely related to atherosclerotic processes (Griggs et al., 1981, Bilora et al., 1999, Methia et al., 2001, van Galen et al., 2012). The amount and structure of plasma VWF are thus important factors in vascular homeostasis.

WPB is a member of the family of lysosome-related organelles (LROs) (Wei and Li, 2013). LRO biogenesis is precisely regulated by HPS (Hermansky-Pudlak syndrome) protein associated complexes (HPACs), such as the BLOC (biogenesis of lysosome-related organelles complex) -1, -2, -3, AP-3 (adaptor protein complex-3) and HOPS (homotypic fusion and protein sorting complex) complexes. Mutations in most of the HPS genes in mice or in humans lead to a bleeding tendency, hypopigmentation and other LRO defects (Wei and Li, 2013). The bleeding diathesis has been linked to platelet granule defects (Wei and Li, 2013, Meng et al., 2015). However, except for AP-3, the functions of these HPACs in WPB biogenesis are largely unclear. Thus despite the importance of endothelial VWF to hemostasis and the differential effects of desmopressin (DDAVP) on HPS patients (Van Dorp et al., 1990, Zatik et al., 2002, Cordova et al., 2005), how the structure and function of VWF and WPB are affected in HPS patients has not been thoroughly established. In this study, we compared DDAVP-triggered VWF secretion into plasma in different HPS mouse mutants with the C56BL/6J background, *pa* (pallid, *Hps9* ^−/−^, BLOC-1 deficiency), *ru* (ruby eye, *Hps6* ^−/−^, BLOC-2 deficiency), and *ep* (pale ear, *Hps1* ^−/−^, BLOC-3 deficiency) and found a range of defects in the stimulated release of VWF multimers that affect both response to DDAVP treatment and hemostasis in HPS patients.

## 2 Results

### 2.1 Stimulated secretion of highly-multimerized (HM) VWF was impaired in *pa, ru,* or *ep* deficient mice

**Fig. 1:**
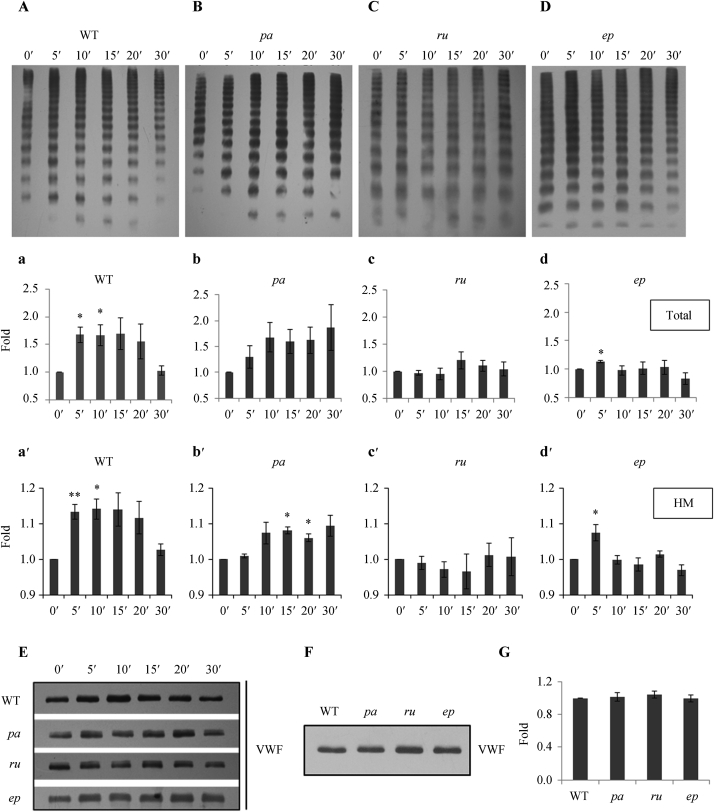
Stimulated secretion of VWF multimers was abnormal in *pa*, *ru* or *ep* mice. **A–D**: Representative Western blots of VWF multimers from single examplar mice are shown. DDAVP (50 μg/kg) was injected into the caudal vein of 12-week-old male mice. After 0, 5, 10, 15, 20 and 30 min, the mouse plasma specimens were collected and the VWF multimers in the plasma of wild-type (WT) mice (**A**), *pa* (BLOC-1 HPS9 deficiency) mice (**B**), *ru* (BLOC-2 HPS6 deficiency) mice (**C**), and *ep* (BLOC-3 HPS1 deficiency) mice (**D**) were analyzed by Western blotting after separation on SDS agarose gels. a–d: The cumulative quantitation of total VWF corresponding to (**A**–**D**) at each time point normalized by that of 0 min are shown. a′–d′: The cumulative quantization of the highly-multimerized (HM) VWF (all VWF upwards from 10 bands from the top) corresponding to (**A**–**D**) at each time point normalized by that of 0 min are shown. Multimer gels were analyzed using NIH Image J software. Stars indicate the significance: *P < 0.05, **P < 0.01; 3 mice were used in each group. **E**: Representative Western blots of increased VWF showed that the total amount of changed plasma VWF after DDAVP stimulation had similar patterns to HM VWF (**A**–**D**). **F**: Representative Western blots of unchanged total VWF in plasma under non-stimulated status of WT, *pa*, *ru* and *ep* mice are shown. **G**: The quantification of total VWF (**F**) in plasma from non-stimulated WT, *pa*, *ru* and *ep* mice (*P* > 0.05). Three mice were used in each group.

The secretion pattern of HM forms of VWF in the plasma immediately after endothelial activation most closely reflects the state of VWF within WPB and thus allows for an indirect assessment of the extent of VWF multimerization within the endothelial cells (ECs) *in vivo* (Nightingale et al., 2009). DDAVP, used to trigger VWF exocytosis in patients (Mannucci, 1997) and in WT (wild-type) mice (Rybaltowski et al., 2011), led to a significant increase in both total and HM VWF by 5 min post-injection, and this increase remained for around 20 min before resolution (Fig. 1A, a and a′). In *pa* mice, the response was delayed by 15 min post-injection (Fig. 1B, b and b′) when a significant (albeit reduced) increase in HM (b′) but not total (b) VWF was seen. In *ru* mice, there was no increase in total (c) or HM (c′) VWF at any time points (Fig. 1C, c and c′). A small increase in total (d) as well as HM (d′) VWF briefly appeared at 5 min in *ep* mice (Fig. 1D, d and d′). Thus, both *ru* and *ep* mice showed very little regulated release of HM VWF. Consistently, in each strain of mouse the total plasma VWF levels in *pa, ru* and *pe* mice changed over time after stimulation in a similar way to the levels of HM VWF present (Fig. 1E). However, the basal VWF levels in WT and mutant mouse plasma were almost the same when compared by immunoblotting (Fig. 1F and G). Furthermore, the secretion patterns of VWF multimers under non-stimulated status (Fig. 1A–D, at each 0′ lane) were almost normal in those three mutant mice. This indicated that only the stimulated secretion of VWF (mainly reflecting the VWF stored in WPBs) but not the basal or constitutive secretion of VWF was affected in *pa, ru*, and *ep* deficient mice. These data suggest that different HPS proteins act differentially in VWF release, implying differential functions in WPB biogenesis and/or exocytosis.

### 2.2 WPBs were misshaped in *ru* mice

**Fig. 2:**
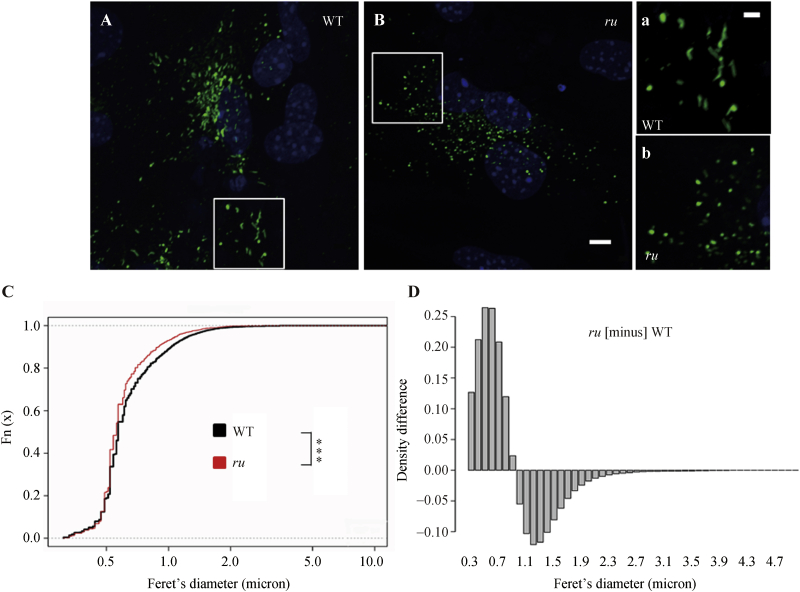
WPBs were misshaped in *ru* mice. **A** and **B**: Representative immunofluorescence images of primary ECs are shown. ECs from WT (**A**) and *ru* (BLOC-2 HPS6 deficiency) (**B**) were isolated from 4-week-old male mice and cultured for 10 days. After immunofluorescence staining of VWF (green), WPBs were examined under a confocal microscope. Typical elongated WPBs in WT ECs were rarer in *ru* ECs. Scale bar: 5 μm. **a** and **b**: The corresponding magnified regions (white boxes) of **A** and **B** showed that WPBs were misshaped in *ru* ECs. Scale bar: 2 μm. **C**: Quantification analysis of the Feret's diameters of WPBs in WT and *ru* ECs. The VWF positive organelles (green in **A** and **B**) whose Feret's diameter is greater than 0.3 μm were counted as WPB. The images were analyzed with NIH Image J software. The distribution of WPBs was plotted as an empirical cumulative distribution as a function of their Feret's diameters. The distribution of WPBs in *ru* ECs shifted towards shorter Feret's diameters compared with that in WT ECs. The total number of WPBs analyzed: 4900 WPBs from WT ECs, 2551 WPBs from *ru* ECs, *** *P* < 0.001. **D**: A differential plot of the Feret's diameter of *ru* WPBs minus that of WT WPBs. There were more WPBs in *ru* ECs than in WT ECs in the positive range, while in the negative range there were less WPBs in *ru* ECs than in WT ECs. More WPBs were distributed in the range of shorter Feret's diameter in *ru* ECs than in WT ECs.

We concentrated further studies on the *ru* mice as showing the most extreme defects in VWF. The altered secretion of HM VWF from cells with defective organelle-forming cellular machinery may result from impaired biogenesis, abnormal exocytosis, or both. As there is HM VWF as well as some basal/constitutive secretion in *ru* mouse plasma (Fig. 1C), this deficiency does not cause a complete failure of formation as in an AP-1 deficiency (Lui-Roberts et al., 2005). Since VWF biosynthesis and WPB morphology are so tightly linked, we determined the morphological characteristics of WPBs in primary ECs isolated from WT and mutant mice. Immunofluorescent staining with VWF antibodies revealed the typical elongated WPBs in WT ECs (Fig. 2A, a), but these were only rarely seen in *ru* ECs (Fig. 2B, b). However, we observed WPB structures similar to those of WT ECs in *pa* or *ep* ECs (Fig. 4). To gain an unbiased comparison of the WPB structure in WT *versus ru* cells, we carried out an automated quantitative morphometric analysis of immunofluorescent images (Ferraro et al., 2014). VWF positive organelles (green) whose Feret's diameter was greater than 0.3 μm were counted as WPBs, and their distribution was plotted (in an empirical cumulative distribution) as a function of Feret's diameter (Fig. 2C). The distribution of WPBs in *ru* ECs shifted towards shorter Feret's diameters compared with that in WT ECs. Using the differential plot for *ru* minus WT (Fig. 2D), we can see that in the positive range there were more WPBs in *ru* ECs than in WT ECs, and in the negative range there were less WPBs in *ru* ECs than in WT ECs. It was confirmed that there were more WPBs distributed in the range of shorter Feret's diameters in *ru* ECs than in WT ECs. This suggests that the WPBs in the ECs from *ru* mice are mostly spherical rather than elongated. Because the tubulation of VWF within WPBs is essential to drive the elongated shape of WPB, such spherical WPBs in *ru* ECs imply that the formation of VWF tubules from VWF multimers may be abnormal in these cells.

### 2.3 WPBs containing organized tubular structure were decreased in *ru* ECs

**Fig. 3:**
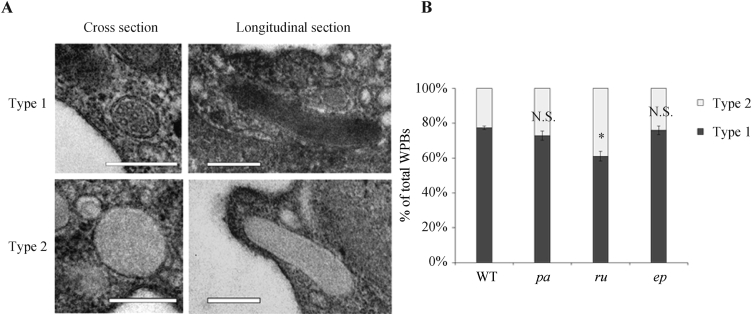
The WPBs containing organized tubular structures were decreased in *ru* ECs. **A**: Representative electron micrographs of WPBs are shown. The aorta was separated from WT and *pa*, *ru* and *ep* mice (12-week-old male mice) and fixed in 2% paraformaldehyde, 2.5% glutaraldehyde and 0.1% tannic acid in 0.1 mol/L phosphate buffer. The representative cross sections and longitudinal sections of type 1 and type 2 WPBs are shown. Type 1 WPBs has highly-ordered tubular structure inside. Type 2 WPBs has unclear and disorganized tubular structure inside. Scale bar: 200 nm. **B**: WPBs of WT and mutant mice were counted, respectively. The total number of WPBs analyzed: 754 WPBs from three WT mice, 754 WPBs from three *pa* mice, 730 WPBs from three *ru* mice, and 794 WPBs from three *ep* mice. The percentages of each type of WPBs in total WPBs are shown. The average percentage of type 1 WPB in *ru* mice (61.0 ± 2.8%) was decreased compared with that in WT mice (77.4 ± 1.0%), in *pa* mice (73.0 ± 2.7%) and in *ep* mice (76.0 ± 2.5%). * *P* < 0.05; N.S., no significance; three mice were used in each group.

To confirm that the misshaped WPBs in *ru* mice do have an impaired tubular structure, we analyzed the ultrastructure of WPBs using transmission electron microscopy (TEM). We defined two types of WPBs (Fig. 3A) in our TEM images from the aorta of WT and mutant mice. Type 1 WPBs have a highly ordered internal tubular structure, whereas type 2 WPBs are internally disorganized. In Fig. 3A, representative cross sections and longitudinal sections of type 1 and type 2 WPBs are respectively shown. Notably, nearly all the WPBs found among the TEM images taken from aorta were cross sections. The longitudinal sections were rarer. The WPBs of WT and mutant mice were categorized and the percentages of each type of WPB are shown in Fig. 3B. The type 1 WPBs were less common in *ru* mice than in controls. In contrast, there were no significant differences in the amounts of these two types in the *pa* and *ep* mice compared with the WT mice. These results are suggestive that the formation of VWF tubular structure is likely impaired in *ru* mice.

### 2.4 Neutralization treatment altered the elongated shape of WPBs in WT, *pa* and *ep* ECs, but not in *ru* ECs

During VWF biosynthesis, dimerization in the ER is followed by further folding in the increasingly acidic environment of the TGN to facilitate the formation of a tubular spiral structure which generates the elongated shape of WPBs, and that also facilitates correct multimerization (Vischer and Wagner, 1994, Huang et al., 2008). The misshaped WPBs and the decrease of organized VWF tubular structures in *ru* ECs seen by EM prompted us to explore this issue further. Treating the ECs with neutralizing reagents such as monensin, NH_4_Cl, or chloroquine, which neutralizes the pH of acidic organelles, results in most WPBs becoming spherical within 1 h (Michaux et al., 2006). The extent of the effect of NH_4_Cl on WPB shape will depend on how their tubular VWF is, i.e., genetically-generated defective folding of VWF will reduce the change in WPB length seen after incubation with NH_4_Cl.

**Fig. 4:**
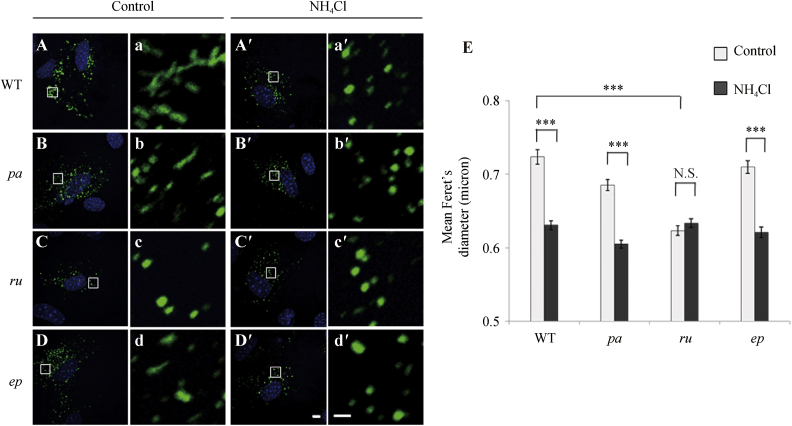
Neutralization treatment altered the elongated shape of WPBs in WT, *pa* and *ep* ECs, but not in *ru* ECs. **A–D**: Representative immunofluorescence images of the primary ECs are shown. Isolated ECs from WT, *pa*, *ru* and *ep* mice (4-week-old male mice) were cultured for 10 days (**A**–**D**) and treated with 20 mmol/L NH_4_Cl in growth medium for 60 min (**A′**–**D′**), and then underwent immunofluorescence staining of VWF (green). Scale bar: 5 μm. a–d: Corresponding magnified regions (white boxes) of **A** to **D**. The WPBs from WT, *pa* and *ep* ECs became round after neutralization treatment (a′, b′ and d′) compared with their elongated shapes before the treatment (a, b and d). While the WPBs in *ru* ECs were round-shaped both before (c) and after (c′) the neutralization treatment. Scale bar: 5 μm. **E**: Quantification of the mean Feret's diameters of WPBs in WT mice and mutant mice before and after NH_4_Cl treatment is shown. The VWF positive organelles (green in **A**–**D**) whose Feret's diameter is above 0.3 μm were counted as WPB. The images were analyzed with NIH Image J software. The total number of WPBs analyzed: *n*_*control*_ = 1 669, *n*_*NH4Cl*_ = 1588 in WT ECs; *n*_*control*_ = 1 799, *n*_*NH4Cl*_ = 1847 in *pa* ECs; *n*_*control*_ = 1 643, *n*_*NH4Cl*_ = 1807 in *ru* ECs; *n*_*control*_ = 1783, *n*_*NH4Cl*_ = 1728 in *ep* ECs. *** *P* < 0.001; N.S., no significance.

To quantify the degree of folding of VWF, we treated the primary ECs from WT and mutant mice with NH_4_Cl. As shown in Fig. 4A and A′, consistent with a previous report (Michaux et al., 2006), the majority of WPBs in WT ECs became round after the neutralization treatment. Similar results were observed in *pa* (Fig. 4 B and B′) and *ep* ECs (Fig. 4D and D′). However, the WPBs in *ru* ECs were already rounded before the neutralization treatment (Fig. 4C and C′). These misshaped WPBs (Fig. 2) were *ru*-specific (not seen in Fig. 4A, B and D, and no change in morphometry in Fig. 4E). These results suggest that HPS6 deficiency likely affects the tubulation of VWF in WPBs, a critical step in VWF biosynthesis.

### 2.5 VWF dispersion and the ability to unfurl into long filaments were impaired in *ru* ECs

**Fig. 5:**
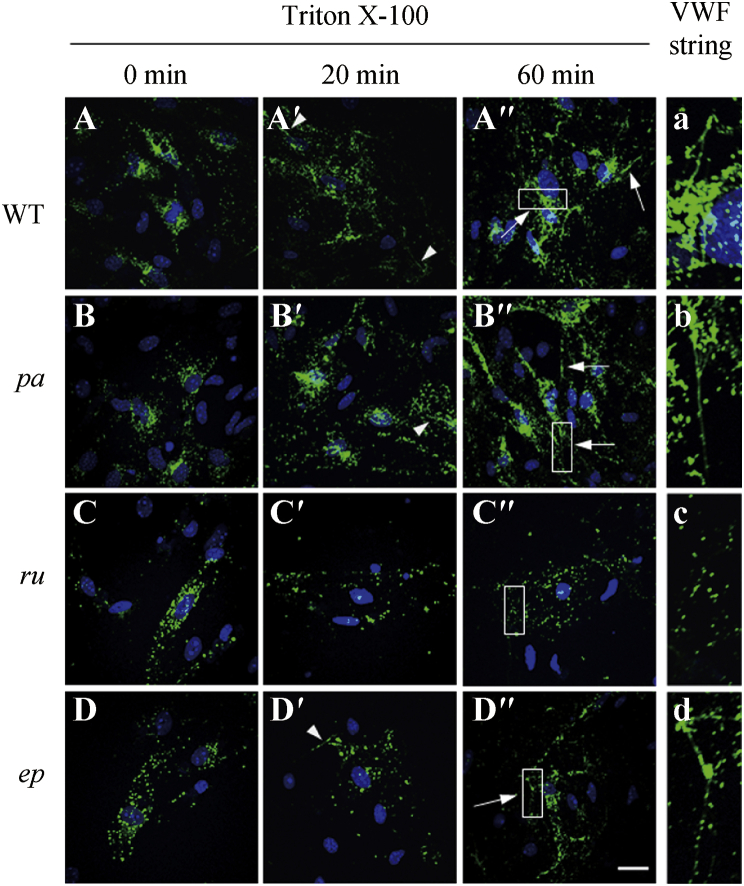
VWF dispersion and the ability to generate large surface structures were affected in *ru* ECs. **A–D**: Representative immunofluorescence images of the primary ECs are shown. ECs from WT, *pa*, *ru* and *ep* mice (4-week-old male mice) were cultured for 10 days (**A**–**D**), then treated with 1% TritonX-100 in growth medium for 20 min (**A′**–**D′**) or 60 min (**A″**–**D″**) on ice. After immunofluorescence staining of VWF (green), the *in situ* dispersion and formation of VWF “blobs” and tangles were observed under a confocal microscope. The arrowheads represent potential classic filaments, occasionally present even though these cells were not exposed to flow. The arrows represent longer filaments. No apparent longer filament was visible in *ru* ECs even after treated for 60 min. Scale bar: 5 μm. a–d: The corresponding magnified regions (white boxes) of **A**–**D** show the longer filaments in WT, *pa* and *ep* ECs but not in *ru* ECs after treated for 60 min.

During exocytosis of WPBs, once facing the neutral pH of plasma, the pro-peptides of VWF dissociate from the mature multimers and the VWF unfurls into long filaments to recruit platelets (Michaux et al., 2006). It has been shown that VWF tubulation is essential for WPB elongation and VWF unfurling (Michaux et al., 2006). To independently confirm that VWF tubulation was impaired in *ru* ECs, the detergent Triton X-100 was used to treat ECs not under flow in neutral pH (pH 7.2) to destroy the membranes surrounding VWF tubules, leaving the VWF multimers to disperse *in situ* when they can form blobs, tangles and even sometimes unfurl into long filaments *in vitro*. As shown in Fig. 5, the dispersion of VWF into clumps, tangles and short filaments appeared at 20 min of detergent treatment in WT, *pa* and *ep* ECs (Fig. 5A′, B′ and D′), and longer filaments were sometimes visible at 60 min (Fig. 5A′′, B′′ and D′′). However, very few smaller structures, let alone the longer filaments, were visible in *ru* ECs during this time period (Fig. 5C′and C′′). This is consistent with the prediction that VWF distribution in the absence of a limiting membrane and their ability to generate long filaments would be altered in *ru* ECs due to the lack of the tubular structures found in mature WPBs. This result again is consistent with defective VWF tubulation in *ru* ECs.

## 3 Discussion

WPB, a member of the LRO family (Wei and Li, 2013), must utilize similar cellular machinery to that of other LROs in their biogenesis. However, while it has been shown that the AP-3 complex is involved in the delivery of CD63 to WPB (Harrison-Lavoie et al., 2006), any specific roles for other HPS protein associated complexes in WPB biogenesis have not been reported. One of the major functions of WPB is to support the formation and storage of HM VWF for the release into blood after secretagogue stimulation. In HPS6 deficient WPBs, VWF tubulation (a critical step in VWF multimerization and in producing VWF that can form blobs, larger tangles and even occasionally unfurl into long strings) (Michaux et al., 2006)) was impaired. Our results demonstrate that HPS6 is required for this aspect of WPB biogenesis, although further details of the underlying molecular mechanism are as yet unknown. One possible explanation might lie in the need for an acidic milieu to support tubule formation. We therefore speculate that HPS6 may affect the delivery of machinery required for acidification of WPB, explaining the failure of NH_4_Cl to significantly alter the shape of the WPB within *ru* ECs. Whether a HPS6 deficiency affects the intra-TGN/WPB pH therefore warrants future investigation.

In this study, the secretion of VWF multimers after DDAVP injection was abnormal with different patterns in HPS9, HPS6 or HPS1 deficient mice compared with that in WT mice. A failure to secrete in response to DDAVP might reflect a reduced pool of untangled and multimerized VWF. VWF in an unfolded state within the WPBs could either fail to multimerize correctly, leaving only low MW materials, or could be multimerized but unable to form tubules, and thus in either case would not be unfurled and stretched out for ADAMTS13 cleavage to proceed, precluding it from joining the usual plasma multimer population. The shapes of WPBs in HPS1 and HPS9 deficient mice are not changed, suggesting that HPS1 or HPS9 likely does not affect the tubulation of WPBs. However, the secretion of VWF after DDAVP stimulation was delayed in HPS9 deficient mice and was very short in HPS1 deficient mice (Fig. 1). The delay of the secretion of high MW VWF multimers in HPS9 deficient mice suggests that the exocytosis of WPB or the unfurling process might be affected. In HPS1 deficient mice, the DDAVP-responsive secretion of HM VWF was very brief. In addition to the kind of problems raised above, further defects could include a smaller readily-releasable pool of mature WPBs, or indeed the accelerated removal of impaired VWF from the circulation. Our data, showing unimpaired levels of fully multimerized VWF in non-stimulated mice (Fig. 1) suggest that at least some multimerization is occurring, even within the *ru* endothelium, and that the loss of a regulated response to DDAVP, the main change seen here, does not affect this. Surprisingly, the loss of elongated WPB shape in *ru* ECs is not associated with a parallel loss of multimerization in plasma, implying that tubulation is not always required for multimerization, or that the population of WPB serving the regulated secretory pathway is selectively defective.

These unexpected and complex phenotypes lead us to conclude not only that there is still a great deal to understand about the packaging and secretion of VWF, but also that the detailed roles of HPS9, HPS6 and HPS1 in VWF biosynthesis, WPB biogenesis or exocytosis, which all require further studies. Our results also suggest not only that these HPS proteins do contribute to the bleeding diathesis of HPS, but also that different HPS proteins act differentially, consistent with the variable patient responses to DDAVP as described below. We therefore propose that the likely altered response to endogenous secretagogue stimulation will differentially contribute to the bleeding tendency in the HPS mice or patients.

It must be emphasized that the steady-state plasma VWF levels of the HPS mutants did not change, but that the changes occurred after DDAVP treatment when multimerized VWF is released for stopping bleeding. While DDAVP has been suggested for the symptomatic treatment of bleeding diathesis in HPS patients (Huizing et al., 2008), variable responses to DDAVP administration in HPS patients (Van Dorp et al., 1990, Zatik et al., 2002, Cordova et al., 2005) have been documented. In particular, 89% of Puerto Rican HPS patients, who are mostly HPS-1 or HPS-3 (forming the same BLOC-2 complex with HPS5 and HPS6 proteins), were reported as poor responses to DDAVP treatment (Cordova et al., 2005). Our results in mice suggest that the administration of DDAVP to the HPS-6 (BLOC-2) or HPS-1 (BLOC-3) subtype patients may well be differentially reduced in efficacy. In the era of precision medicine, these findings suggest that genotyping of HPS patients might be explicative of the results of DDAVP administration.

Interestingly, the *ru* mutant showed a protective role in atherosclerosis after an atherogenic diet, while the *ep* mice had lesions and reduced life span similar to control C57BL/6J mice. In addition, the atherosclerotic lesions did not correlate with the platelet serotonin levels (Paigen et al., 1990). It has been shown that VWF deficiency or loss of function has a protective effect on atherosclerosis (van Galen et al., 2012). These studies suggest that other factors such as VWF may be responsible for the variability of susceptibility to atherosclerosis in these mouse HPS mutants. The defects in the secretion of HM VWF upon DDAVP treatment in the *ru* mice could confer the resistance for atherosclerosis in these mice or HPS6 patients.

## 4 Materials and methods

### 4.1 Mice

*pa*, *ru*, *ep* mutant mice (*pa* – HPS9 deficiency in BLOC-1 (Huang et al., 1999), *ru* – HPS6 deficiency in BLOC-2 (Zhang et al., 2003), *ep* – HPS1 deficiency in BLOC-3 (Gardner et al., 1997)) and the control C57BL/6J mice (WT) were originally obtained from The Jackson Laboratory (Maine, USA) that were maintained in Dr. Richard T. Swank's laboratory. All these mutants arose from spontaneous mutations in C57BL/6J background. These mice were bred in the animal facility of the Institute of Genetics and Developmental Biology (IGDB), Chinese Academy of Sciences. The mutant mice were maintained as homozygotes through intercrosses of heterozygotes. The genotypes of these mutants were confirmed by PCR genotyping methods based on the nature of the mutations (Li et al., 2006). Male mice of each genotype were used to avoid potentially confounding hormonal effects of the estrous cycle in females. All animal procedures were approved by the Institutional Animal Care and Use Committee of IGDB.

### 4.2 Isolation of primary endothelial cells (ECs)

Primary ECs were isolated as previously described (Jin et al., 2012). Dynabeads coated with Sheep anti-Rat IgG (Invitrogen, catalog# 110.35, USA) were pre-treated by incubating the beads with Rat anti-mouse PECAM-1 (CD31) antibody (BD Biosciences, catalog# 553370, USA) at 4°C overnight, washing four times with PBS + 0.1% BSA and then resuspending beads in PBS + 0.1% BSA. Two weeks old male mice were euthanized by injection of sodium pentobarbital and dipped in 70% ethanol for 5 min. The heart was removed and minced, then incubated in 0.2% collagenase type II (Invitrogen, catalog# 17101-015) with Hank's buffered salt solution (HBSS: dissolve 200 mg of KCl, 30 mg of KH_2_PO_4_, 175 mg of NaHCO_3_, 4 g of NaCl, 24 mg of Na_2_HPO_4_, 500 mg D-glucose, and 3 g HEPES in 500 mL distilled water; adjust pH to 7.4) at 37°C with gentle rotation for 45 min. The suspension was filtered through a 70-μm disposable cell strainer (Corning, catalog# 352350, USA) and spun at 400 × *g* for 8 min at 4°C. The cells were resuspended in DMEM and incubated with 20 μL/mL pre-treated anti-mouse PECAM-1 coated Dynabeads for 30 min at 4°C. Beads were then washed 3–5 times until supernatant is clear, then incubated with 0.05% trypsin-EDTA at 37°C for 5 min before removal of the beads. Cells were then spun down at 400 × *g* for 10 min, and the cell pellet was resuspended in growth medium and plated in a 6-well plate.

### 4.3 Cell culture and treatments

Mouse primary ECs were grown in EBM-2 medium (Lonza, catalog# CC-4176, USA) at 37°C in 5% CO_2_. NH_4_Cl (Sigma-Aldrich, catalog# A9434, USA) was used in 20 mmol/L final concentration in growth medium. Triton X-100 (Sigma-Aldrich, catalog# T8787) was used at 1% in PBS after pH adjustment. The Triton X-100 treated cells were applied on ice for 20 min or 60 min. All the procedures were followed as described (Michaux et al., 2006).

### 4.4 Antibodies and immunofluorescence imaging

Polyclonal rabbit anti-human VWF antibody was purchased from DAKO (Carpinteria, catalog# A0082, USA). Immunofluorescence staining and confocal microscopy were carried out as previously described (Zhang et al., 2014). Cells were grown on glass cover slips in 24-well plates and fixed with freshly prepared 4% paraformaldehyde for 30 min, washed three times with 0.01 mol/L phosphate buffer (PBS, pH 7.4), permeabilized with 0.3% Triton X-100 in PBS for 15 min, and blocked in 0.01 mol/L PBS containing 1% BSA for 1 h. Fixed cells were incubated with anti-VWF antibodies (1:2000 in PBS containing 1% BSA) overnight at 4°C, then washed three times with 0.01 mol/L PBS containing 0.1% Triton X-100 before incubating with Alexa Fluor 488-conjugated secondary antibody (Life technologies, catalog# A21206, USA) for 1 h at room temperature. Then the glass cover slips were mounted with Mounting Medium with DAPI (ZSGB, catalog# ZLI-9557, China). Images were acquired with a 100× objective of ECLIPSE Ti-C2 confocal microscope (Nikon, Japan). The images were analyzed with NIH Image J software. Morphometric analysis of images was previously described (Ferraro et al., 2014).

### 4.5 VWF multimer analysis

DDAVP (Sigma-Aldrich, catalog# V1005) powder was dissolved in 0.9% NaCl solution. DDAVP (20 ng/μL) was injected into the caudal vein of 12-week-old male mice at a dose of 50 μg/kg. After 0, 5, 10, 15, 20 and 30 min, the tail blood of mouse at each time point were collected and centrifuged at 4000 × *g* for 10 min. The supernatant plasma was collected for immunoblotting.

VWF multimer analysis was previously described (Nightingale et al., 2009). 1.2% agarose gels were prepared by dissolving Seakem high gelling temperature agarose (Lonza, catalog# 50041) in 0.375 mol/L Tris (pH 8.8) with SDS added to a final concentration of 0.1%. Mouse plasma samples (3 μL) were loaded in 50 mmol/L Tris pH 8.0, 1% SDS, 5% glycerol and 0.002% bromophenol blue. Gels were run at 30 V for 16 h (Beijing Liu Yi, China) before transfer to a nitrocellulose membrane, labeled with rabbit anti-VWF antibody followed by a HRP conjugated anti-rabbit secondary antibody (ZSGB, catalog# ZB-2301, China) and developed by chemiluminescence (SuperSignal West Pico, Thermo, catalog# NCI5080, USA). The multimers of each lane on the same gel were arranged according to the MW. We chose the top 10 bands as a cutoff for quantification of high MW since there is no universally accepted multimer level above which they are high MW as against low MW, we therefore used a pragmatic solution and chose the highest multimer at which we can be sure of matching the bands between samples. Multimer gels were analyzed using NIH Image J software.

### 4.6 Electron microscopy

Mice were euthanized by injection of sodium pentobarbital and dipped in 70% ethanol for 5 min. The thoracic cavity was incised, the sternum cut, and the aorta cleaned by perfusing with fixing buffer (2% paraformaldehyde and 2.5% glutaraldehyde) from the left ventricle. The aorta was separated gently and the surrounding connective tissue and fat were removed, the vessel was cut into 1 mm tubes, and fixed in 2% paraformaldehyde, 2.5% glutaraldehyde and 0.1% tannic acid in 0.1 mol/L phosphate buffer at 4°C overnight. After washing, the tubes were post-fixed in 1% osmium tetroxide for 1 h, dehydrated in an ascending series of dilution of acetone and impregnated in Spurr. Blocks were polymerized at 70°C for 12 h, sections (60 nm) cut with a Leica microtome (Ultracut, Germany), stained with lead citrate, and viewed with a JEM 1400 electron microscope (JEOL, Japan).

### 4.7 Statistical analysis

The data are shown as the mean ± S.E.M. Comparisons were statistically tested using the Student's *t*-test. Difference of *P* < 0.05 was considered as statistically significant. For blinded quantification, an automated unbiased high-throughput computer-driven morphometric methodology was used and unknown genotypes were not informative to the observers.
